# Observations on Mr. Cornish's Account of a Convulsive Epidemic in Cornwall

**Published:** 1814-06

**Authors:** James M'Donald

**Affiliations:** City Road


*For the Medical and Physical Journal.*

### *Observations on Mr. Cornish's Account of a Convulsive Epidemic in Cornwall; by* Mr. James M'Donald.

HAVING recently published in your Journal an article signed James Cornish, purporting to be an historical account of a "Convulsive Epidemic," said to be extremely prevalent in some parts of Cornwall, you will, it is hoped, be so candid as to insert the following brief remarks upon that curious production.

Your correspondent assumes, as an incontrovertible truth, that "religious enthusiasm has been the exciting cause" of what he considers a bodily disease of a most contagious nature, which, notwithstanding the violence of the symptoms, has not, to his knowledge, proved mortal in a single instance. Nor does he hint that recourse has been had, in any case, to medical assistance. It must of course be a most singular "convulsive epidemic," a phenomenon for future inquiry, by which, in a short space, six thousand persons, of different ages and complexions, have been attacked without sustaining any material injury. Nor is the "principle of imitation," through the medium of which Mr. C. maintains the contagion has spread far and wide with "the rapidity of lightning," less inexplicable. Why preaching, similar to that which the people at Redruth heard at the commencement of the "convulsive epidemic" there, does not produce similar effects in every town and village throughout the United Kingdom, even when the weather is not fair, nor the air dry or cold, to counteract its influence, remains to be accounted for. If what Mr. C. terms "religious enthusiasm," and "the principle of imitation," be such powerful agents among thousands of the inhabitants of Cornwall, upon what principle are we to account for their being inactive in any place where they are found among men of like passions?

And

And if, from an *individual* crying out in a congregation, "What must I do to be saved?" such "a convulsive epidemic" has been produced as has affected *thousands*, it will be found no easy task for the faculty to point out a quarantine sufficiently strict to prevent the universal spread of the contagion.

"The sect," saith Mr. C. "with whom this singular affection originated, holding an opinion that the Spirit of God operates sensibly in the conversion of men, ascribe this work to divine agency; and in proof of this opinion, adduce as an analogous instance the conversion of three thousand men, under the influence of a sermon preached them by the apostle Peter. When to this is opposed the improbability that the Divine Spirit should confine its operations to the chapels of their particular sect, they reply, that 'God works how and where he pleases.'" The methodists believe, with the primitive church, and that of England, and assign reasons for their doing so, that the conversion of sinners is effected by the agency of the Divine Spirit, and that the effects consequent on genuine conversion, such as divine "love, joy, and peace," are perceived by all those who have them in their possession; and that the existence of those effects cannot be accounted for upon natural principles. Such as wish to know their reasons for thus believing, I refer to the Rev. Mr. Wesley's Appeals, by the perusal of which some philosophic infidels have been led to embrace christianity. In short, the methodists believe their Bible, and consequently that "it is God who worketh in men both to will and to do of his good pleasure." But if by the Divine Spirit "working *sensibly* in the conversion of men" be meant any thing more than that the effects produced by such agency are discernible by, and evident in, those in whom they are wrought, they reject with abhorrence such a gross notion of the divine operation. Nor do they believe that the Divine Spirit confines his operations to their chapels, nor that, in order to the conversion of sinners, any such distress of mind, and consequent bodily symptoms, as are described by Mr. Cornish, are necessary. The symptoms in question, they well know, are mere circumstances which may be thoroughly accounted for on rational and scriptural principles; and that they cannot, with the colour of reason, be attributed either to enthusiasm or "the principle of imitation." That God works "how and where he pleases," is a truth to which men of the first attainments have not scrupled to subscribe.

Most of the symptoms which Mr. C. ascribes to "religious enthusiasm," and "the principle of imitation," are represented by the Church of England, in the *Homily on fasting*, as as accompanying true repentance. Witness her words:—"When men feel in themselves the heavy burden of sin, see damnation to be the reward of it, and behold with the eye of their mind the horror of hell; they tremble, they quake, and are inwardly touched with sorrowfulness of heart, and cannot but accuse themselves, and open their grief unto Almighty God, and call upon him for mercy. This being done seriously, their mind is so occupied, partly with sorrow and heaviness, partly with an earnest desire to be delivered from hell and damnation, that all desire of meat and drink is laid apart, and loathsomeness (or loathing) of all worldly things and pleasure, cometh in its place. So that nothing then liketh them, more than to weep, to lament, to mourn, and both with words and behaviour of body to shew that they are weary of life."

To what would a philosopher, who denies the agency of the Divine Spirit in the conversion of men, attribute the symptoms so clearly and forcibly described in this quotation? Doubtless to "religious enthusiasm," and "the principle of imitation." But if they are justly attributable to those principles, what a set of enthusiastic men were our learned and venerable reformers; and how powerfully does "the principle of imitation" (as a medium for conducting "a convulsive epidemic") now work on the members of the Homily Society!

"When," saith Mr. C. "the strength is nearly exhausted, the patients usually swoon, and they remain in a rigid and motionless state, until respite from fatigue, fresh air, or some more powerful stimulus, enables them to re-act, or to put a stop to the curious character they had undertaken." (p. 376.) Here it is left doubtful whether the patients, as they are called, were voluntary or involuntary agents; but if the former, it is most unaccountable how thousands of different ages, complexions, and mental endowments, should, without any previous instruction or motive whatever, by an act of their will, bring upon themselves a distressing paroxysm sufficient to stop respiration. How much more difficult is it to believe this, than to admit the powerful agency of the Divine Spirit upon their minds, though some of the effects produced should to us remain incomprehensible! That such effects are not usually produced is no reason why we should attribute them to a cause which reason, common sense, and experience, demonstrate to be incapable of producing them.

That no immoral character, when known to be such, is permitted to remain in the methodist society, is well known to all who are acquainted with their economy. It follows, upon upon Mr. Cornish's hypothesis, that about six thousand individuals, a majority of whom were grossly immoral a few months ago, have been recently reclaimed from their vicious habits by a "convulsive epidemic." A disease, so salutary in its effects, would not be injurious to the metropolis. But to be serious:—That a bodily disease should produce such an amazing change as has recently taken place in thousands of the inhabitants of Cornwall, is abundantly more difficult of belief than all the miracles recorded in the Scriptures. For let the doctrine of the being and attributes of God be admitted, and it will follow that all things which do not imply a contradiction are possible to him; and surely it implies no contradiction that he in whom we live, move, and have our being, should, in a manner beyond our contracted grasp, work a moral change conducive to their happiness, in thousands of his rational offspring. To make the unusualness of the method by which such a change may have been effected in any person an argument against God's being the author of it, seems to be as unreasonable as to maintain that pestilence and earthquakes, on account of their being uncommon, are neither subject to his controul, nor under his direction.

Without supposing that Mr. C. has intentionally misrepresented any of the facts recorded in his paper, it is proper to observe, that three accounts which now lie before me, written by eye-witnesses, who are men of sound sense and strict integrity, differ materially from that which he has given. This being the case, your readers, who are in general men of science, will, I trust, suspend their censure till they have an opportunity of judging, from the moral effects of the supposed "convulsive epidemic," to what cause it ought to be attributed.

JAMES M'DONALD.

*City Road, April* 27, 1814.

